# Low-Cycle Fatigue Properties of Bimetallic Steel Bar with Buckling: Energy-Based Numerical and Experimental Investigations

**DOI:** 10.3390/ma17163974

**Published:** 2024-08-09

**Authors:** Xuanyi Xue, Fei Wang, Neng Wang, Jianmin Hua, Wenjie Deng

**Affiliations:** 1 School of Civil Engineering, Chongqing University, Chongqing 400045, China; xuexuanyi@cqu.edu.cn (X.X.); 27130461@alu.cqu.edu.cn (F.W.); huajianmin@cqu.edu.cn (J.H.); dengwenjie@stu.cqu.edu.cn (W.D.); 2 Key Laboratory of New Technology for Construction of Cities in Mountain Area, Ministry of Education, Chongqing University, Chongqing 400045, China; 3 School of Management Science and Real Estate, Chongqing University, Chongqing 400045, China

**Keywords:** stainless-clad bimetallic steel bar, fatigue performance, dissipated energy density, buckling mode, numerical simulation

## Abstract

A bimetallic steel bar (BSB) consisting of stainless-steel cladding and carbon steel substrate exhibits excellent corrosion resistance and good mechanical properties. The bimetallic structure of BSBs may affect their low-cycle fatigue performance, and current investigations on the above issue are limited. In this study, the low-cycle fatigue properties of bimetallic steel bars (BSBs) with inelastic buckling were investigated. Experiments and numerical studies were conducted to investigate the low-cycle fatigue capacity for BSBs, considering buckling. The buckling mode of BSBs is discussed. The hysteretic loops and energy properties of BSBs with various slenderness ratios (*L*/*D*) and fatigue strain amplitudes (*ε_a_*) are investigated. With increases in the *L*/*D* and *ε_a_*, the original symmetry for hysteresis loops disappears gradually, which is caused by the buckling. A predictive equation revealing the relation between the *ε_a_* and fatigue life is suggested, which considers the effects of the *L*/*D*. A numerical modelling method is suggested to predict the hysteretic curves of BSBs. The effect of buckling on the stress and energy properties of BSBs is discussed through the numerical analysis of 44 models including the effects of the *L*/*D*, *ε_a_*, and cladding ratios. The numerical analysis results illustrate that the hysteresis loops of BSBs with various *ε_a_* values exhibit similar shapes. The increase in the cladding ratio reduces the peak stress and the dissipated energy properties of BSBs. The hysteresis loop energy density decreases by about 3% with an increase of 0.1 in the cladding ratio. It is recommended that the proportion of stainless steel inBSBs should be minimized once the corrosion resistance requirements are met.

## 1. Introduction

Corrosion is one of the important factors affecting the durability of reinforced concrete structures [1,2,3]. The alkaline environment in the concrete protects the steel reinforcement from corrosion by the passivation layer. However, with an increase in service time, the gradual carbonation of the concrete directly reduces the pH value of the internal environment of the concrete [4,5,6]. With the deepening of carbonation, the passivation layer on the steel reinforcement surface gradually disappears [7]. The steel reinforcement undergoes corrosion when it comes in contact with corrosion factors in the environment [8]. Corrosion oxidizes the iron element in the steel reinforcement, which negatively alters the dimensions of the steel reinforcement [9,10]. Corrosion products change the bond performance between the steel reinforcement and concrete [11,12,13]. The high degree of corrosion considerably reduces the bonding performance [14,15]. With an increase in the service time of existing reinforced concrete structures, the adverse effects of the corrosion gradually become significant. Thus, the impact of corrosion on reinforced concrete structures must be urgently solved or mitigated. Notably, stainless steel exhibits excellent corrosion resistance [16,17]. In contrast, carbon steel has excellent mechanical properties, but is susceptible to corrosion. However, stainless steel is considerably more costly than carbon steel; therefore, the use of carbon steel reinforcement in reinforced concrete structures is more prevalent than that of stainless-steel reinforcement. If the advantages of stainless and carbon steels can be combined to form a material with superior corrosion resistance and mechanical properties, and a lower cost than stainless-steel reinforcement, the durability of reinforced concrete structures can be improved. With the development of metal materials science, bimetallic materials, such as titanium-clad bimetallic steel [18,19,20], bimetallic steel bars (BSB) [21], and stainless-clad bimetallic steel [22,23], have gradually entered the field of vision of researchers. The BSB consists of cladding and substrates, which are stainless and carbon steels, respectively. The stainless steel insulates the carbon steel from corrosion factors, thereby avoiding corrosion problems. Through the hot-rolling process, a stable metallurgical bonding layer is formed between the stainless and carbon steels; this layer ensures synergistic deformation between the cladding and substrate. Hua et al. [24] investigated the tensile performance of BSBs. The experimental results indicated that the performance of BSBs satisfied the requirements of the current design code, and their excellent ductility was observed. Furthermore, the bond properties of BSBs in concrete have been experimentally investigated [25]. Based on the above investigations, BSBs are believed to have broad application prospects in reinforced concrete structures that require outstanding durability. 

When reinforced concrete structures are subjected to earthquakes, their load-bearing members are often subjected to cyclic loads [26]. Plastic hinges occur at the beam–column joints. Previous studies have shown that longitudinal steel reinforcements in the plastic hinge region often exhibit fatigue fracture failure [27,28]. The large cyclic deformation generated by an earthquake results in the failure of steel bars within 1000 cycles, which is referred to as low-cycle fatigue (LCF) damage. Numerous researchers have investigated the LCF performance of structural steel. Based on the dissipated energy property, Abdalla et al. [28] proposed prediction methods for the LCF life of BS 460B/B500B steel reinforcements. Apostolopoulos and Papadopoulos [29] experimentally investigated the LCF properties of S400 steel reinforcements, and the effects of corrosion were considered. Caprili and Salvatore [30] selected different steel reinforcements to perform a fatigue experiment wherein cyclic behaviour was introduced. Stirrups can limit the transverse deformation of concrete and steel bars. For a reinforced concrete structure under an earthquake, the longitudinal reinforcement between stirrups may perform inelastic buckling [31]. The spacing of the stirrups affects the effective length of longitudinal steel bars and directly influences their inelastic buckling behaviour. Inelastic buckling causes deformation concentration, which accelerates the accumulation of fatigue damage. Subsequently, inelastic buckling reduces the LCF performance of the reinforcements [32]. Similar to the buckling of steel columns, the buckling of steel bars reduces the compressive bearing capacity. In general, to accurately evaluate the hysteretic behaviour of load-bearing members, the effect of buckling on the LCF behaviour of steel bars must be investigated. Current investigations on the above issue are limited and have focused primarily on carbon steel reinforcement. Tripathi et al. [33] evaluated the effect of buckling on the LCF life of reinforcements. Aldabagh and Alam [34] experimentally studied the LCF life and energy properties of reinforcements with inelastic buckling. Previous research has focused on ordinary carbon steel reinforcement, and the study of inelastic buckling for BSBs was limited. It is worth noting that the bimetallic structure of BSBs may affect the LCF performance and accumulation of LCF damage in the region of the plastic deformation concentration. Therefore, the impacts of buckling on the LCF behaviour of BSBs must be investigated through experiments and numerical methods. In Section 2, an experiment including BSB specimens with various slenderness ratios is performed. The LCF behaviour and failure mode are also discussed. In Section 3, a numerical modelling method is proposed to simulate the cyclic stress–strain curves of BSB specimens under LCF. The effects of the cladding ratio (*β*), slenderness ratio (*L*/*D*), and fatigue strain amplitude (*ε_a_*) on the hysteretic properties of the BSBs are studied using the numerical modelling method, where the stress and dissipated energy properties are included.

## 2. Low-Cycle Fatigue Experiment

### 2.1. Materials

**Figure 1:**
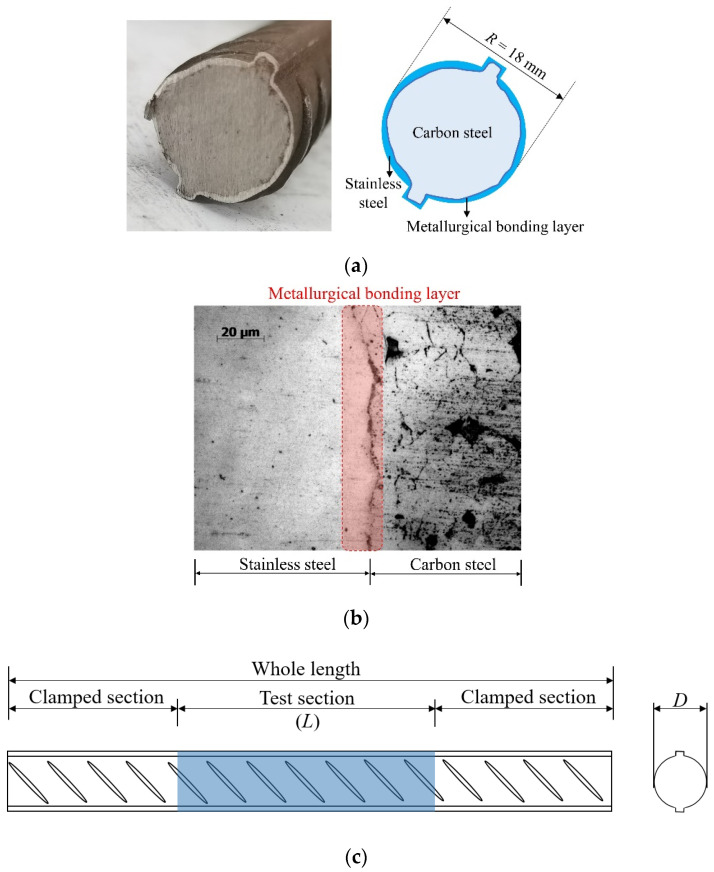
Details of BSBs: (**a**) cross section; (**b**) metallurgy structure [35]; (**c**) specimen design.

**Figure 2:**
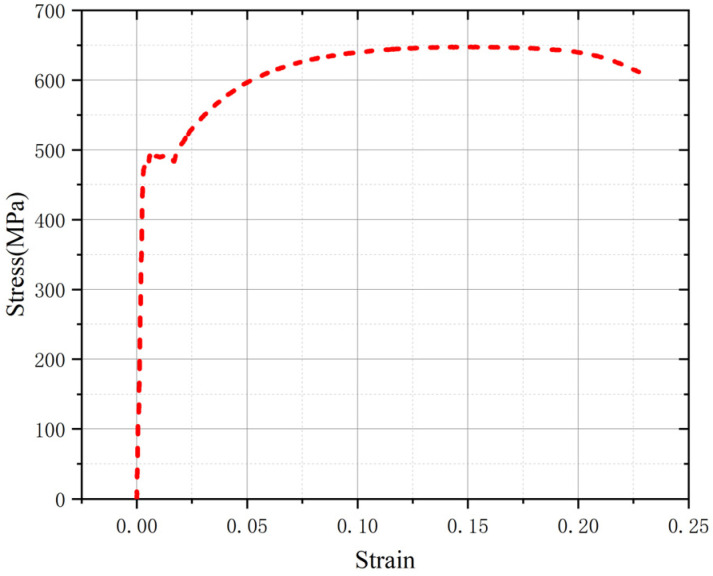
Stress–strain property of BSBs.

The structural form of a BSB is shown in Figure 1a, where the cladding metal is S30408 stainless steel and the substrate metal is HRB400 carbon steel. The chemical compositions of the cladding and substrate materials are listed in Table 1. As shown in Figure 1b, a metallurgical bonding layer formed between the cladding and substrate ensured collaborative deformation. Flake pearlite and ferrite were distributed in the carbon steel area. The BSB was produced by a hot-rolling process. The mean values of the thickness of the cladding and substrate were 0.7 and 8.3 mm, respectively. The dimensions of the LCF specimens are presented in Figure 1c and Table 2. The slenderness ratio is an important parameter in structural design which has a significant impact on member stability, load-bearing capacity, and deformation performance. The larger the slenderness ratio of a member, the more likely it is to buckle. In this study, different slenderness ratios (*L*/*D*) were considered to verify the effects of buckling on the LCF property, where *D* denotes the diameter of the specimen and *L* denotes the length of the specimen test section. LCF specimens with *L*/*D* values ranging from 6 to 15 were prepared. The stress–strain property of the BSBs is shown in Figure 2. There is a clear yield plateau after the elastic segment. Strain hardening was observed before the peak point. After the peak point, a gentle descent segment occurs. In general, the stress–strain properties of BSBs are similar to that of mild steel, rather than that of stainless steel. 

### 2.2. Test Methods

**Figure 3:**
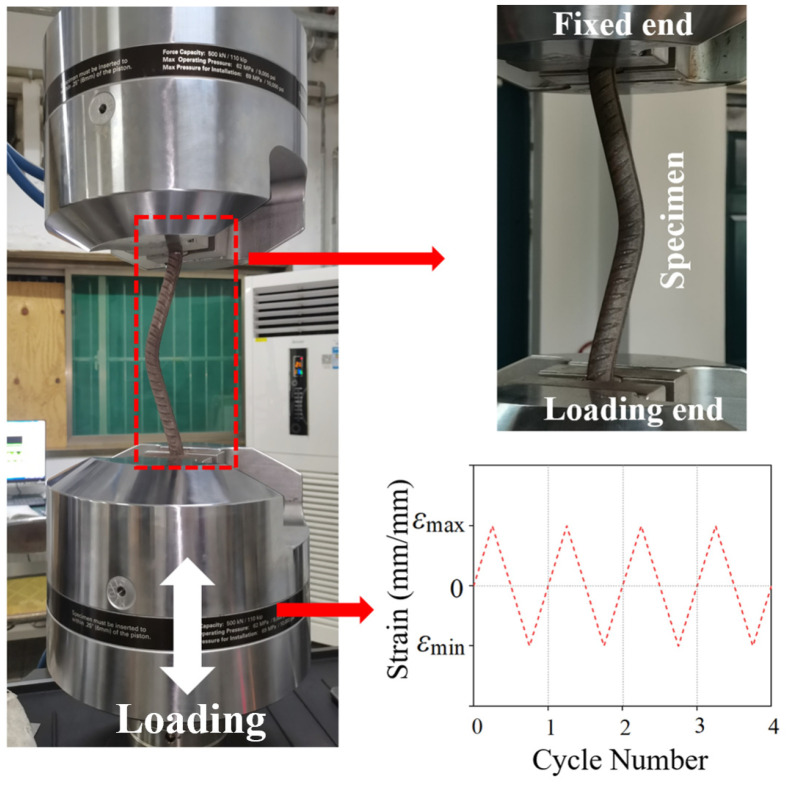
Fatigue loading system.

To apply the LCF load, the MTS Landmark 370.50 testing system (Mechanical Testing & Simulation, Eden Prairie, MN, USA) was selected in this study, as shown in Figure 3. During the installation, the test specimens were debugged to ensure the consistency of the length of the test section with the design. To meet the requirements of GB/T 26077-2021 [36], a vertical reciprocating load was applied to the specimen. In the concrete structure, the vertical reinforcement was constrained by stirrups and concrete, and its two ends can be fixed. Therefore, the top and bottom of the BSB specimen were clamped by fixtures to simulate the above boundary conditions. The *ε_a_* values of the tension and compression were the same, which indicated that the strain ratio was −1. Considering the significant effect of the strain amplitude on the LCF properties of reinforcing and structural steels, the strain amplitude was predetermined as 0.01, 0.02, and 0.03. Three repeated specimens were set for each specimen size and strain amplitude. Based on the investigation conducted in reference [37], frequencies of loading change from 0 Hz to 10 Hz were used to mimic those seen in real earthquakes. Therefore, the test was conducted at a constant strain loading rate of 0.01 s^−1^ to ensure a loading frequency between 0 Hz and 10 Hz.

### 2.3. Buckling Mode

**Figure 4:**
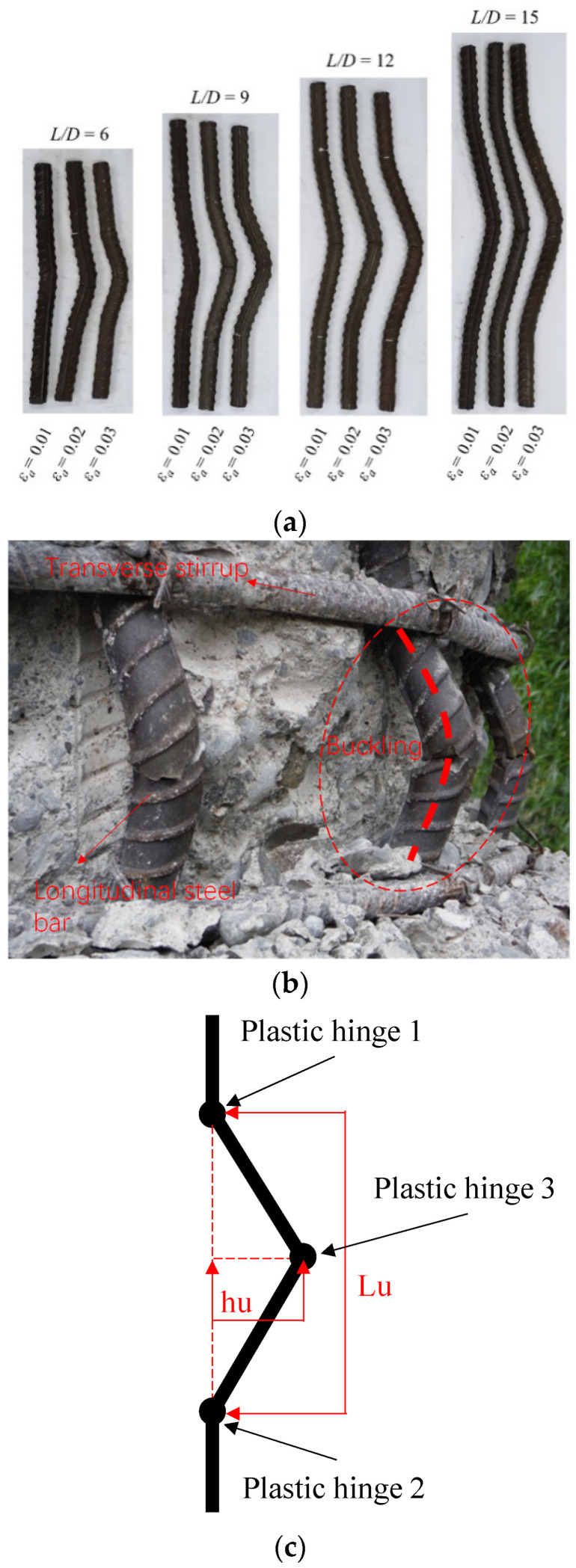
Buckling mode in fatigue: (**a**) buckling mode of BSBs with various *ε_a_* and *L*/*D*; (**b**) buckling of longitudinal reinforcement between stirrups [31]; (**c**) typical buckling mode of BSB specimen.

**Figure 5:**
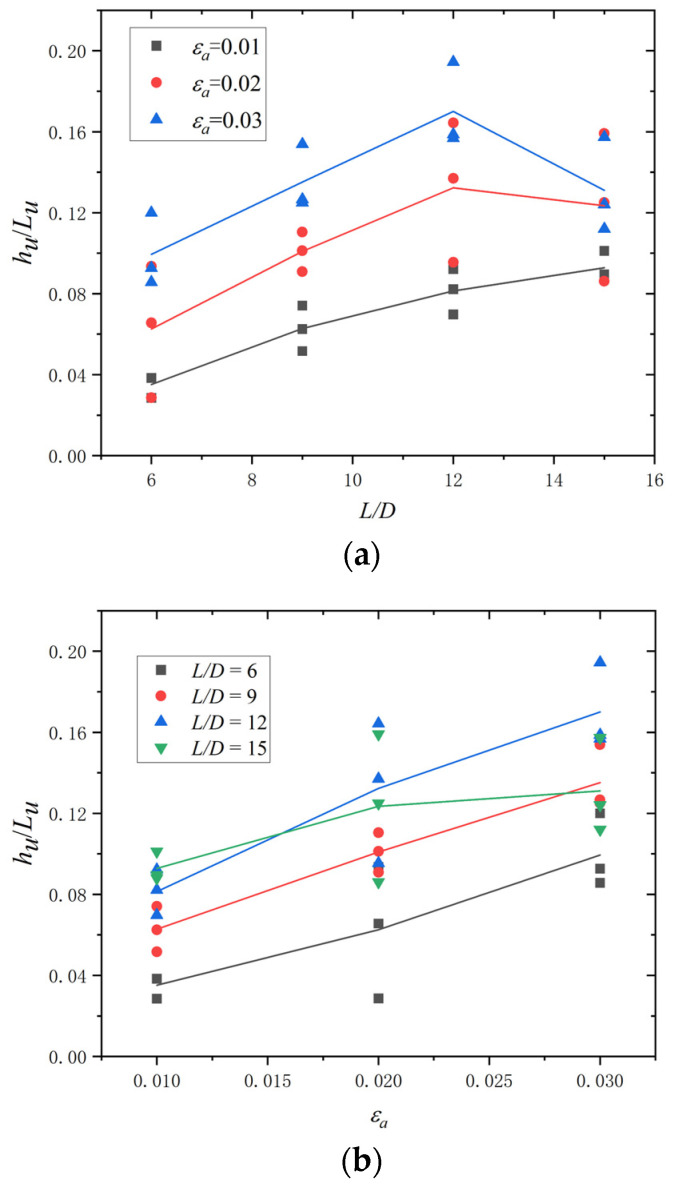
Variation trends of the *h_u_*/*L_u_*: (**a**) *h_u_*/*L_u_* vs. *L*/*D*; (**b**) *h_u_*/*L_u_* vs. *ε_a_.*

**Figure 6:**
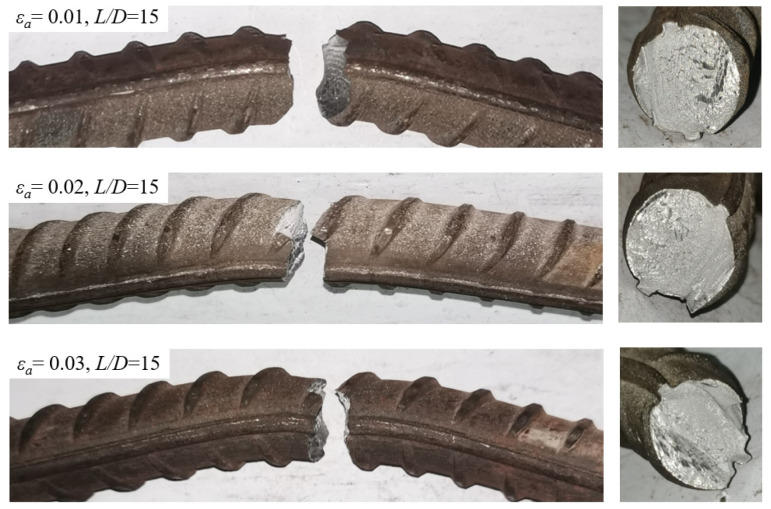
Fracture of BSB specimens.

After the fatigue test, an inelastic buckling mode was recorded for all BSB specimens, as shown in Figure 4a. It can be seen that the buckling mode of the BSBs was similar to the buckling mode of longitudinal bars under earthquakes (Figure 4b). As the *ε_a_* and *L*/*D* increased, buckling was further evident. Plastic hinges with buckling were observed (Figure 4c). Plastic hinges 1 and 2 were distributed at both ends of the test section, whereas plastic hinge 3 was distributed in the middle of the test section. The transverse deflection *h_u_* of plastic hinge 3 and the distance *L_u_* between plastic hinges 1 and 2 were recorded. The dimensionless ratio *h_u_*/*L_u_* was selected to effectively describe the inelastic buckling mode of the BSB specimens. The variation trends of the *h_u_*/*L_u_* for the different specimens are shown in Figure 5. Evidently, the *L*/*D* and *ε_a_* significantly affected the *h_u_*/*L_u_*. When *ε_a_* = 0.01, with an increase in the *L*/*D*, the *h_u_*/*L_u_* increased gradually. For BSB specimens with *ε_a_* = 0.02 and 0.03, with an increase in the *L*/*D*, the *h_u_*/*L_u_* first increased and then decreased, with the turning point being *L*/*D* = 12. It is worth noting that the increase in the *L*/*D* was achieved by increasing the *L*, resulting in a significant increase in *L_u_*. However, the change in *h_u_* was not significant compared to the increase in *L*. The above phenomenon resulted in the *h_u_*/*L_u_* of the specimen with an *L*/*D* of 15 being smaller than that of the specimen with an *L*/*D* of 12. For BSB specimens with the same *L*/*D*, the *h_u_*/*L_u_* increased gradually with an increase in *ε_a_*. Figure 6 shows the fracture morphologies of the BSB specimens. It can be seen that, even after evident buckling deformation, the cladding and substrate were closely connected, and there was no obvious bulge, cracking, or falling-off in the stainless-steel cladding. It is interesting to note that the scanning electron microscopy (SEM) technology is worth applying to further investigate the fatigue fracture mechanism of BSBs.

### 2.4. Test Results and Discussion

**Figure 7:**
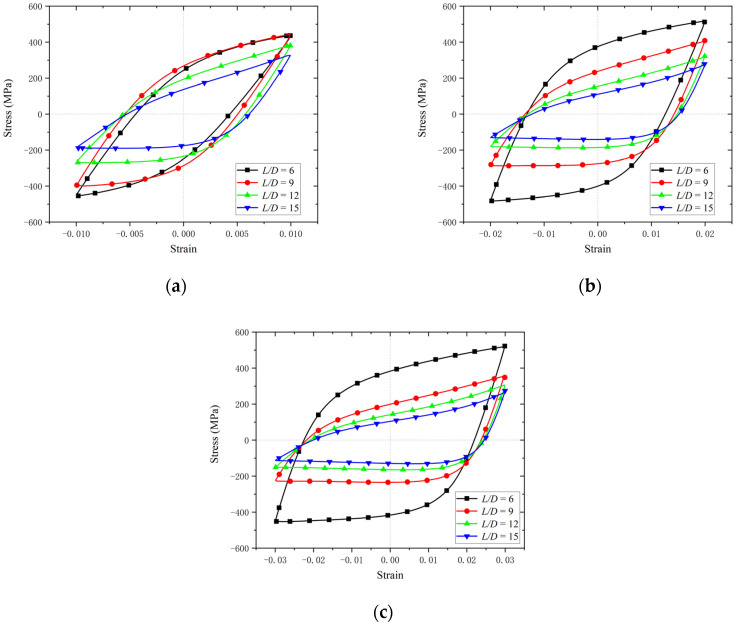
Stable hysteresis loop with different strain amplitudes: (**a**) 0.01; (**b**) 0.02; (**c**) 0.03.

**Figure 8:**
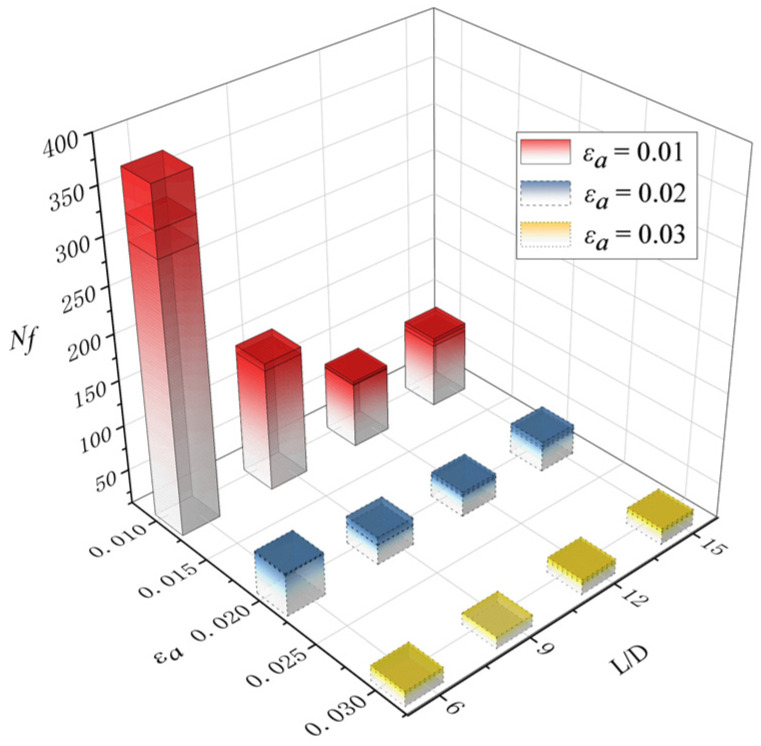
Influences of *ε_a_* and *L*/*D* on cycles to failure.

After fatigue loading, the hysteretic curves and fatigue life of the BSBs were collected. To clarify the impacts of the *L*/*D* and *ε_a_*, a hysteresis loop corresponding to 50% of the LCF life was selected for comparison (Figure 7). The hysteresis loop was shuttle type, which indicated that the BSBs have good plastic deformation ability, excellent seismic performance, and energy dissipation capacity. For the BSB specimen with *L*/*D* = 6 and *ε_a_* = 0.01, no buckling was observed and the hysteresis loop with *L*/*D* = 6 and *ε_a_* = 0.01 exhibited original symmetry. As shown in Table 3, the peak compressive stress *σ*_c,max_ gradually became smaller than the peak tensile stress *σ*_t,max_ with increases in the *L*/*D*. The original symmetry of the hysteresis loop gradually disappeared owing to buckling. For a relatively large *L*/*D*, the BSB specimen under compressive load tended to undergo buckling, which was similar to the case of the steel column under compressive load. The specimen exhibited a significant decrease in stress when it was subjected to compression. The local pinching observed in this hysteresis curve was a typical characteristic of the hysteresis curves of BSB specimens affected by buckling. The buckling resistance of a steel column under a compressive load can be determined by Equation (1), where *N_E_* is the buckling resistance, *E* is the elastic modulus of structural steel, *I* is the area moment of inertia, and *l* is the effective length [38]. The increase in *l* adversely affected the buckling resistance of the steel column under a compressive load. Considering that the buckling of the column was unstable, the *σ*_c,max_ of the BSB specimen was reduced owing to buckling. With an increase in the *L*/*D*, the *l* gradually increased. Hence, owing to the increases in the *L*/*D*, the BSBs became more prone to buckling (Equation (1)). Therefore, the increases in the *L*/*D* adversely affected the *σ*_c,max_ of the BSBs. Notably, for the BSB specimens under the same *ε_a_*, with increases in the *L*/*D*, the *σ*_c,max_ and *σ*_t,max_ of the BSB specimens gradually decreased. Buckling reduced the *σ*_c,max_ of the specimen, leading to serious fatigue damage at the plastic hinge position. Therefore, the *σ*_t,max_ of the specimen also showed a significant decrease. In conclusion, an increase in *L*/*D* will significantly weaken both the *σ*_c,max_ and *σ*_t,max_ of BSBs. Considering that there was no buckling for the BSB specimen under tensile load, the aforementioned decrease in *σ*_t,max_ was caused by fatigue damage; more details are introduced in [39]. Based on the test results, both the *L*/*D* and *ε_a_* had considerable effects on the cycles to failure of the BSBs (Figure 8). With increases in the *L*/*D* and *ε_a_*, the cycles to failure decreased. For the specimens with various *L*/*D* values, the effects of the *ε_a_* on the cycles to failure changed gradually. Therefore, the *L*/*D* and *ε_a_* exhibited a coupling effect on the cycles to failure.

(1) $$N_{E}=\pi^{2}EI/l^{2}$$

**Figure 9:**
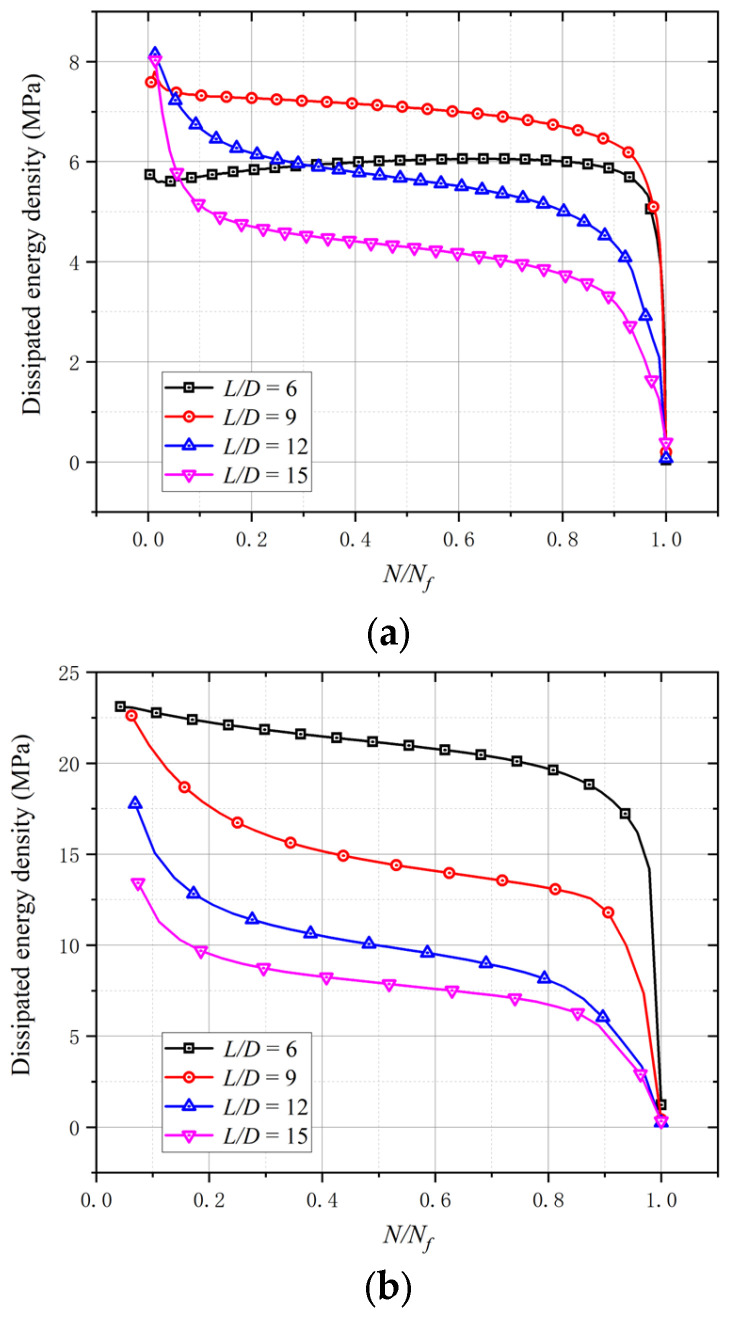

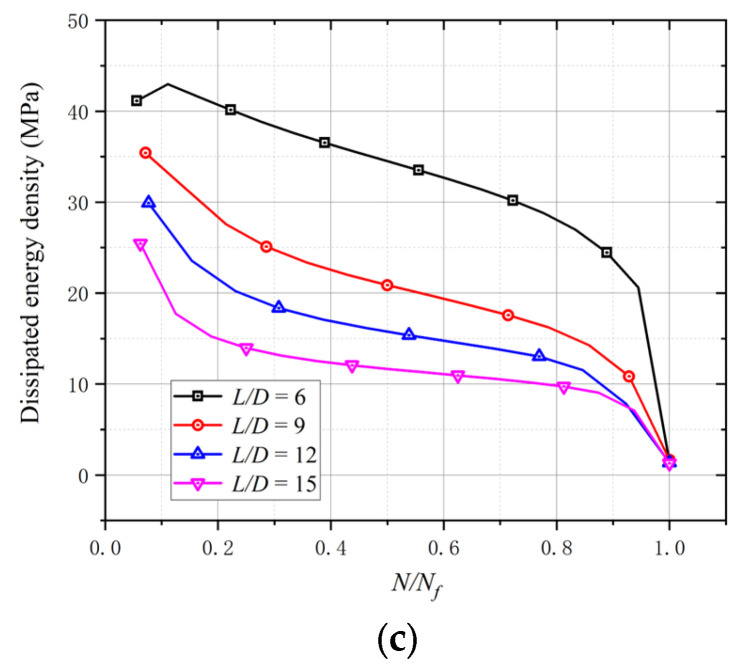
Influences of *L*/*D* on dissipated energy density with different strain amplitudes: (**a**) 0.01; (**b**) 0.02; (**c**) 0.03.

**Figure 10:**
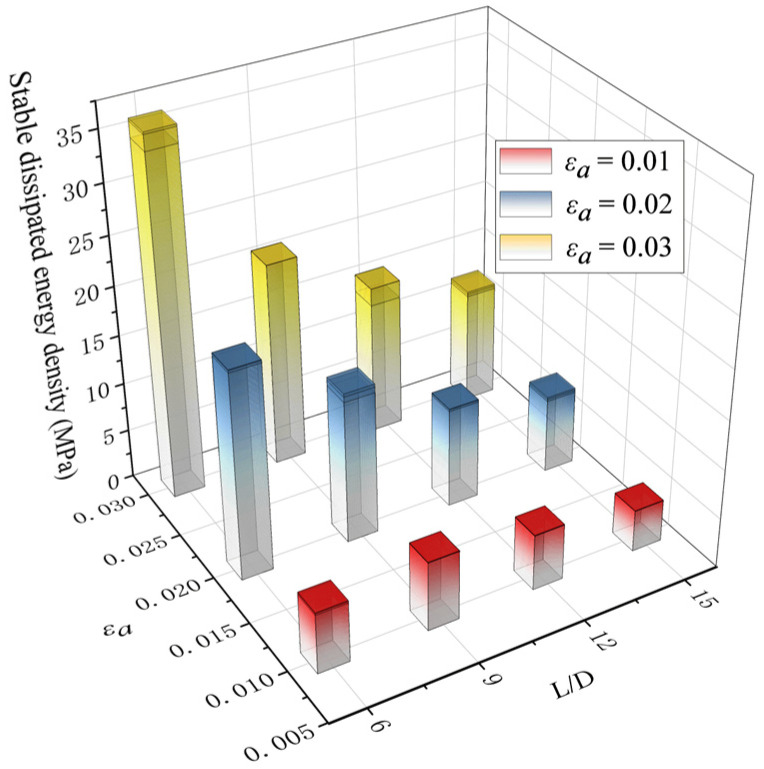
Influences of *ε_a_* and *L*/*D* on stable dissipated energy density.

To clarify the LCF properties of BSBs, dissipated energy density, which is the envelope area of the hysteresis loop, was selected. The area enclosed by the hysteresis curve of BSBs reflects the energy dissipation capacity of the BSB. The area of the hysteresis curve has become an important indicator for evaluating the mechanical properties of materials. The larger the envelope area of the hysteresis curve, the better the plasticity and toughness of the material. After the test, the relationships between the *N*/*N_f_* and dissipated energy density were obtained (Figure 9), where *N* and *N_f_* are the loading cycle and cycles to failure, respectively. Except for specimens with *L*/*D* = 6 and *ε_a_* = 0.01, the dissipated energy density decreased gradually with an increasing *N*/*N_f_*. When the *N*/*N_f_* was relatively small, a clear reduction in the initial segment of the dissipated energy density curve was observed. Subsequently, a relatively stable segment was observed. Next, an obvious reduction was observed when *N* was near *N_f_*. The two inflection points of the above curves corresponded to *N*/*N_f_* = 0.2 and 0.8. When *ε_a_* equaled 0.02 and 0.03, increases in the *L*/*D* decreased the dissipated energy density. The variation trend of the stable dissipated energy density corresponding to 50% *N_f_* is shown in Figure 10. When *ε_a_* = 0.01, with an increase in the *L*/*D*, the stable dissipated energy density first increased and then decreased; the turning point was *L*/*D* = 9. Although the *σ*_c,max_ and *σ*_t,max_ values of specimens with *L*/*D* = 6 were larger than those of BSBs with *L*/*D* = 9, the plastic strain performances of specimens with *L*/*D* = 6 were poorer than those of BSBs with *L*/*D* = 9, which was the reason why the stable dissipation energy density of specimens with *L*/*D* = 9 was higher than that of specimens with *L*/*D* = 6. When *ε_a_* equaled 0.02 and 0.03, decreases in the *L*/*D* beneficially affected the stable dissipated energy density. 

**Figure 11:**
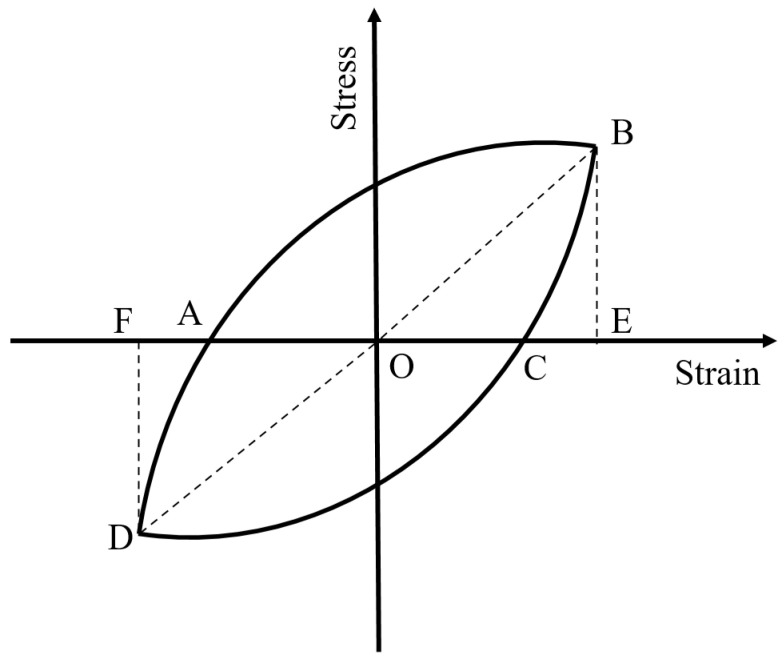
Energy dissipation coefficient.

**Figure 12:**
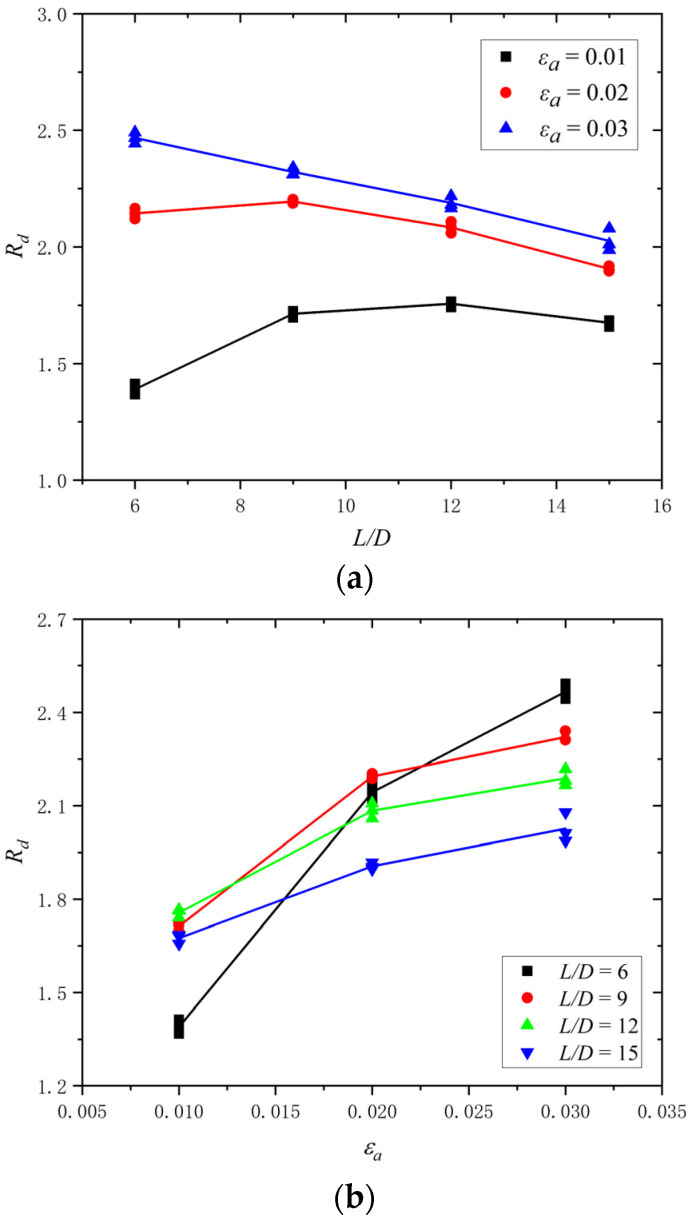
*R_d_* of BSB specimens: (**a**) *R_d_* vs. *L*/*D*; (**b**) *R_d_* vs. *ε_a_.*

Based on the above investigation, buckling was proved to significantly affect the energy dissipation capacity of the BSBs. The energy dissipation coefficient *R_d_* is an important index for quantifying the energy dissipation capacity of a material. It was selected to reveal the effect of buckling. The *R_d_* was calculated using Equation (2), where *S*_(*ABCD*)_ denotes the envelope area of the hysteretic loop and *S*_(*OBE*+*ODF*)_ denotes the area of triangles OBE and ODF (Figure 11). The BSB specimen with a larger *R_d_* exhibited better energy dissipation capacity. Based on the test results, the variation trend curves of *R_d_* corresponding to a stable hysteretic loop with 50% *N_f_* are shown in Figure 12. For BSBs with various *L*/*D* (*ε_a_*) values, the influences of *ε_a_* (*L*/*D*) on *R_d_* were different, and their coupling influence was observed. For BSB specimens with *ε_a_* = 0.02 and 0.03, with increases in the *L*/*D*, the *R_d_* of the BSB specimens first increased and then decreased. For the BSB specimens with *ε_a_* = 0.01, with increases in the *L*/*D*, the *R_d_* decreased gradually, and a negative linear correlation was observed. With increases in *ε_a_*, the *R_d_* of the BSBs with various *L*/*D* values increased gradually. In general, to accurately quantify the LCF behaviour of BSBs, the effects of the *L*/*D* and *ε_a_* must be taken into account.

*R_d_* = *S*_(*ABCD*)_/*S*_(*OBE*+*ODF*)_ (2)

## 3. Numerical Model of BSB Specimen

### 3.1. Modelling Method

**Figure 13:**
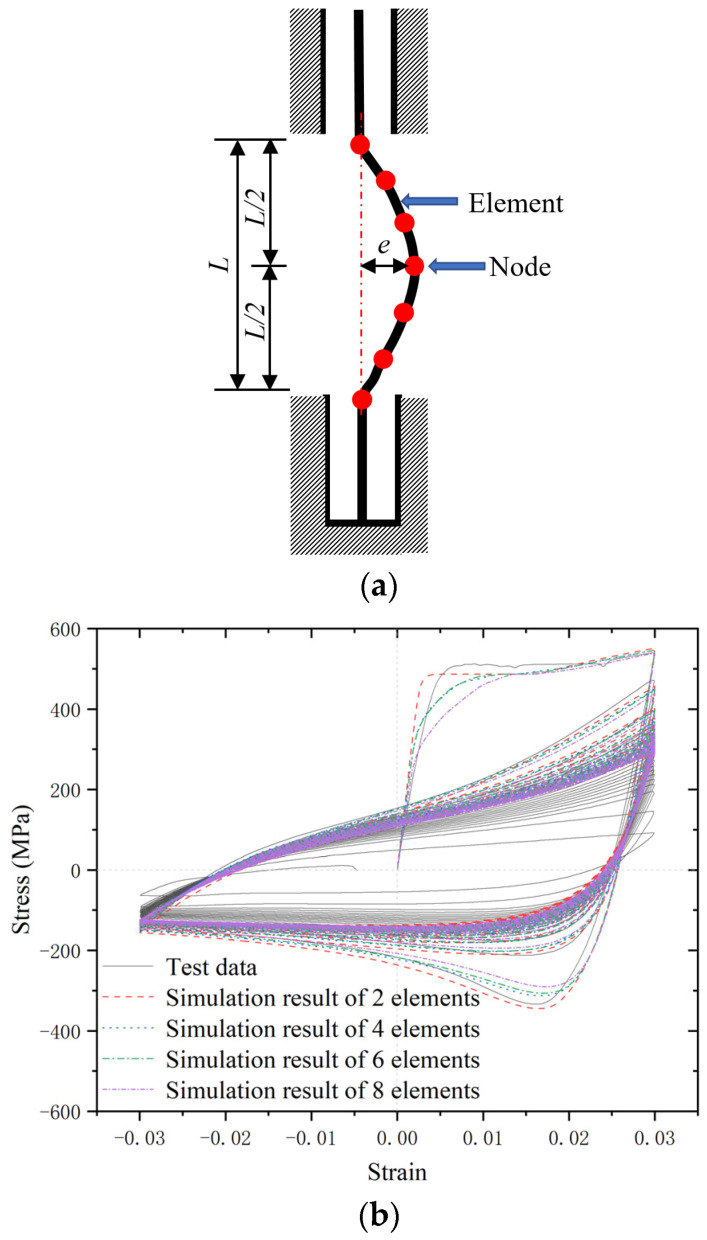
Numerical model of BSB specimens: (**a**) distribution of elements; (**b**) comparison of simulation results of different element numbers.

The LCF properties of BSBs, considering the buckling effect, were simulated using OpenSees (version 3.3.0 64 Bit). As shown in Figure 13a, the numerical model comprises nodes and elements. The size of the numerical model was the same as that of the BSB specimens. The effects of the buckling were introduced by pre-adding the initial geometric defects. The specimen was pre-added with an initial geometric eccentricity (*e*) at the midpoint, where *e* = *L*/1000. The sine function image was considered as the initial imperfection shape of the numerical model, and the node position was assigned. To study the effects of the element number on the simulation results, BSB models with different element numbers were simulated and compared with the test results, as shown in Figure 13b. The effect of the element numbers on the simulation results was found to be negligible. Therefore, a BSB model with two elements and three nodes was adopted in this study to reduce calculated resource consumption. The reinforcing steel material model in OpenSees was selected to describe the material properties of BSBs, which were obtained from reference [40]. The reinforcing steel material model simulated the anisotropic hardening of materials by moving the skeleton curve and adjusting the inflection points of the skeleton curve. During the simulation of the fatigue loading process, the bottom of the BSB model was fixed, and a vertical equal-amplitude displacement load was applied to the top part of the BSB model.

### 3.2. Validation and Discussion

**Figure 14:**
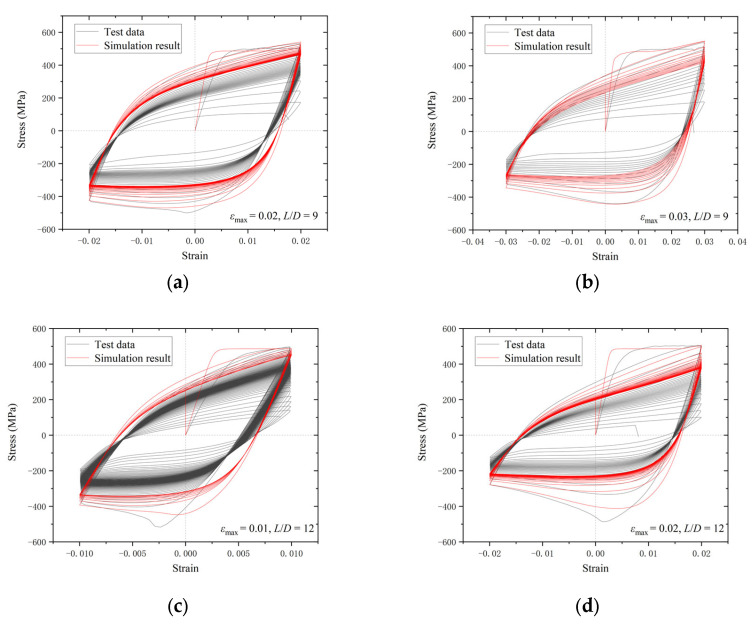

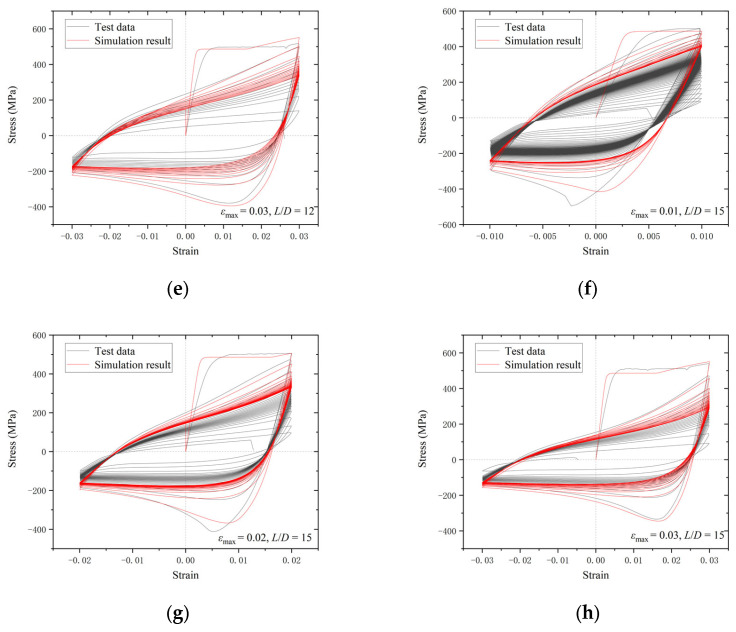
Comparison between test and simulated results: (**a**) *ε*_max_ = 0.02, *L*/*D* = 9; (**b**) *ε*_max_ = 0.03, *L*/*D* = 9; (**c**) *ε*_max_ = 0.01, *L*/*D* = 12; (**d**) *ε*_max_ = 0.02, *L*/*D* = 12; (**e**) *ε*_max_ = 0.03, *L*/*D* = 12; (**f**) *ε*_max_ = 0.01, *L*/*D* = 15; (**g**) *ε*_max_ = 0.02, *L*/*D* = 15; (**h**) *ε*_max_ = 0.03, *L*/*D* = 15.

**Figure 15:**
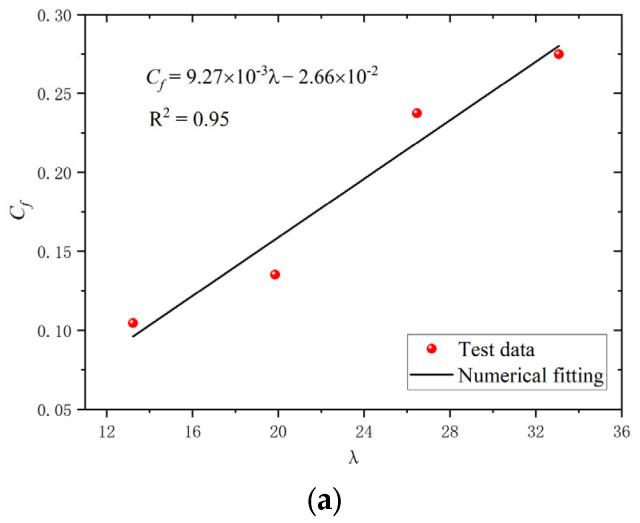

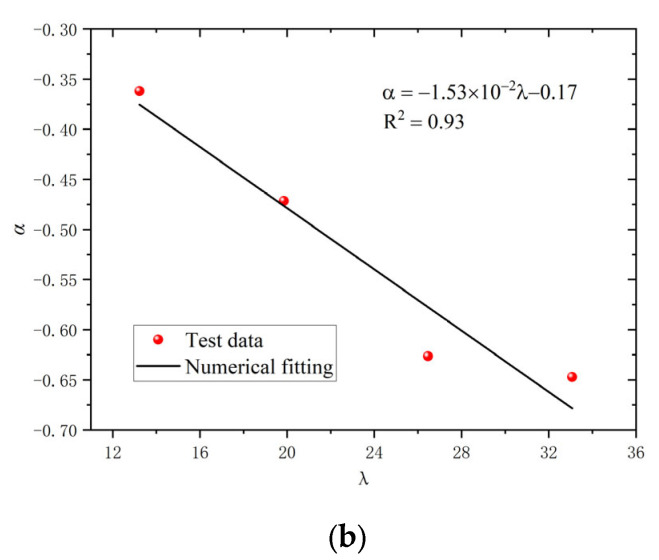
Fatigue model coefficient: (**a**) *C_f_*; (**b**) *α*.

**Figure 16:**
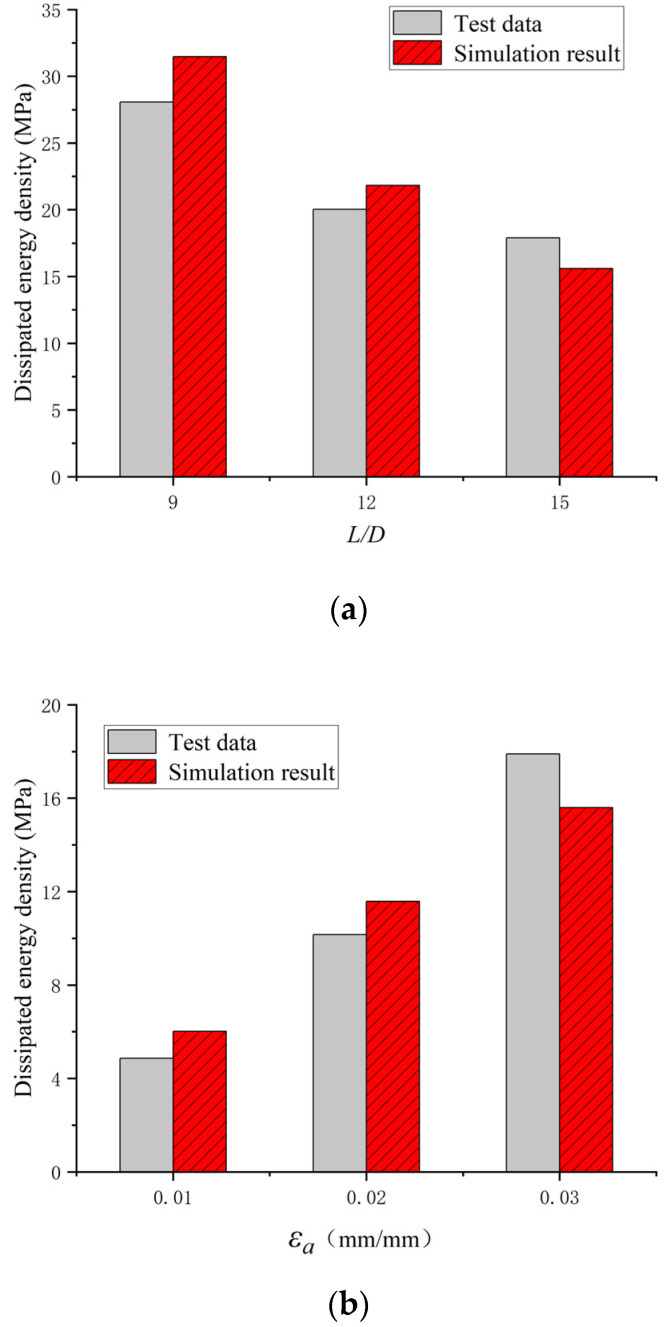
Comparison between the dissipated energy density values of the hysteretic loop corresponding to 20% *N_f_* for the test and numerical results: (**a**) *ε_a_* = 0.03; (**b**) *L*/*D* = 15.

The cyclic curves between the test and numerical results for the BSBs with various *L*/*D* and *ε_a_* values were compared to validate the accuracy of the numerical modelling method (Figure 14). The shape of the hysteretic loop was properly simulated using a numerical model. The asymmetry of the *σ*_c,max_ and *σ*_t,max_, caused by buckling, could be simulated using the numerical model. When *N*/*N_f_* was relatively small, the error between the test and numerical results was negligible. For the condition *N*/*N_f_* = 0.2, the hysteretic loops of the BSBs were relatively stabilized. Hence, the numerical model proposed in this study was used to investigate the hysteretic loop and dissipated energy properties of the BSB corresponding to *N*/*N_f_* = 0.2. Equations (3)–(6) were suggested to predict the *N_f_* of BSBs with various *L*/*D* and *ε_a_* values, which quantified the coupling influences of the *L*/*D* and *ε_a_* on *N_f_* introduced in Section 2.4. A relation between *ε_a_* and *N_f_* in Equation (3) has been suggested by several researchers [29,40,41]. Based on the test results, the *C_f_* and *α* values of the BSBs were obtained (Figure 15). It can be considered that there is a strong linear relationship between λ and the coefficient. To include the effects of buckling, *λ*, determined by Equation (6), was considered in Equations (4) and (5). After numerical fitting, the coefficients *C_f_* and *α* were determined using Equations (4) and (5), respectively. Subsequently, Equation (3) was used to predict the cycles to failure of the BSBs, including buckling influences. The dissipated energy density of the hysteretic loop corresponding to 20% *N_f_* was compared between the test and numerical results (Figure 16). Evidently, the dissipated energy density of the BSBs could be accurately predicted using the numerical model. Next, in Section 3.3, a parameter study was conducted to comprehensively reveal the dissipated energy properties of BSBs with various *L*/*D* and *ε_a_* values.

(3) $$\varepsilon_{a}=C_{f}{\left(2N_{f}\right)}^{\alpha }$$

(4) $$C_{f}=9.27\times 10^{-3}\lambda -2.66\times 10^{-2}$$

(5) $$\alpha =-1.53\times 10^{-2}\lambda -0.17$$

(6) $$\lambda =\frac{L}{D}\sqrt{\frac{f_{y}}{100}}$$

### 3.3. Effect of Slenderness Ratio and Fatigue Strain Amplitude

**Figure 17:**
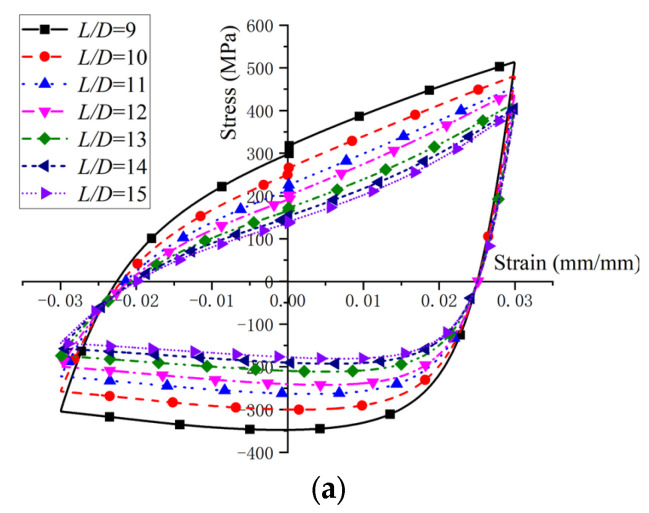

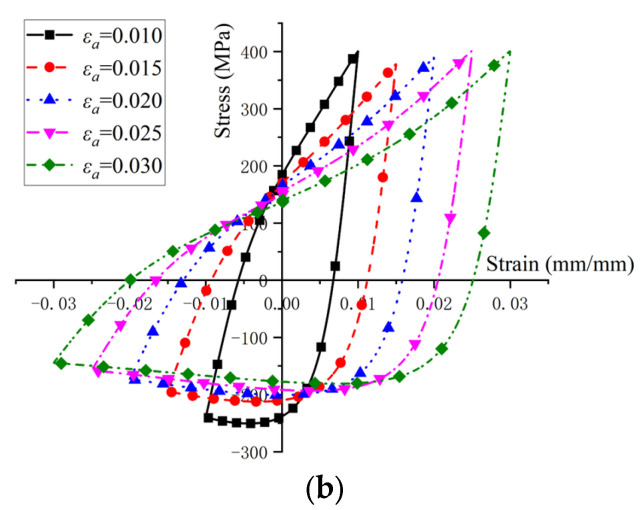
Hysteretic loop corresponding to 20% *N_f_*: (**a**) *ε_a_* = 0.03; (**b**) *L*/*D* = 15.

**Figure 18:**
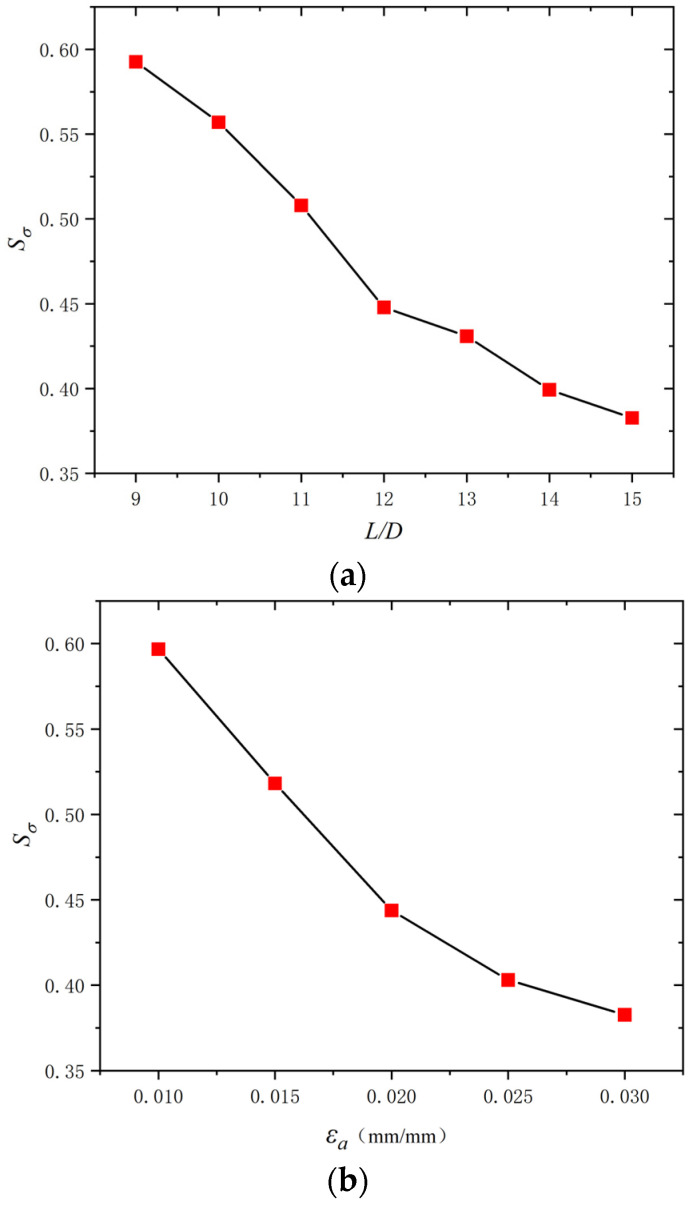
Influences of *L*/*D* and *ε_a_* on *S_σ_*: (**a**) *ε_a_* = 0.03; (**b**) *L*/*D* = 15.

**Figure 19:**
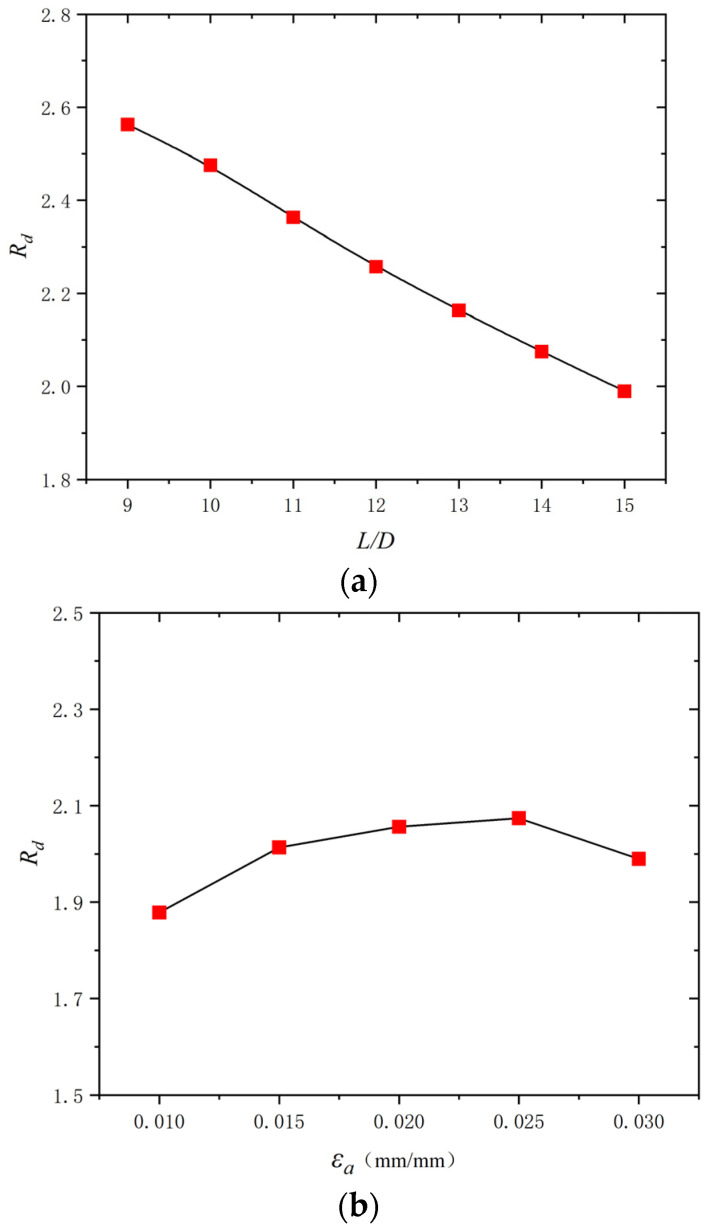
Effects of *L*/*D* and *ε_a_* on *R_d_*: (**a**) *ε_a_* = 0.03; (**b**) *L*/*D* = 15.

Considering the significant effect of the *L*/*D* and *ε_a_* on the dissipated energy properties of BSBs, a parameter study focused on these factors was conducted through the numerical model in Section 3.1. The hysteretic loops corresponding to 20% *N_f_* of the BSBs, based on the numerical analysis, are shown in Figure 17. The *N_f_* value of the BSB model was determined using Equation (3). The *σ*_c,max_ was lower than the *σ*_p,max_ caused by buckling. As the *L*/*D* increased, the pinching phenomenon of the hysteresis loop gradually became evident. The *σ*_c,max_ and *σ*_p,max_ were reduced with an increasing *L*/*D*. The factor *S_σ_* was selected to quantify the above issue and was determined using Equation (7), where $\sigma_{peak}^{-}$ and $\sigma_{peak}^{+}$ are the *σ*_c,max_ and *σ*_p,max_, respectively. Based on the test results, the variation trend of *S_σ_* for BSBs with various *L*/*D* value was collected (Figure 18). With increases in the *L*/*D*, the *S_σ_* gradually decreased, and a linear relationship between the *L*/*D* and *S_σ_* was observed. For BSBs with various *ε_a_* values, the hysteretic loops corresponding to 20% *N_f_* exhibited a similar shape. The effects of *ε_a_* on the *σ*_p,max_ were ignorable. With increases in *ε_a_*, the *σ*_c,max_ gradually decreased. After calculation, the *S_σ_* values for BSBs with various *ε_a_* values are shown in Figure 18. As *ε_a_* increased, the *S_σ_* of the BSBs decreased almost linearly. The impacts of the *L*/*D* and *ε_a_* on *R_d_* are shown in Figure 19 to reveal the dissipated energy properties of the BSBs. As the *L*/*D* increased, the *R_d_* of the BSBs decreased linearly. Hence, the increase in *L*/*D* exacerbated the buckling, which adversely affected the dissipated energy properties. When *ε_a_* ranged from 0.01 to 0.03, the *R_d_* of the BSB first increased and then decreased gradually. The increase in *ε_a_* resulted in a larger plastic strain, which increased the *R_d_*. However, the incremease in *ε_a_* exacerbated the buckling. When the *ε_a_* was 0.03, the adverse effects of buckling were greater than the beneficial effects of the plastic strain on the *R_d_*, which reduced the *R_d_*.

(7) $$S_{\sigma }=\frac{\sigma_{peak}^{-}}{\sigma_{peak}^{+}}$$

### 3.4. Effect of Cladding Ratio

**Figure 20:**
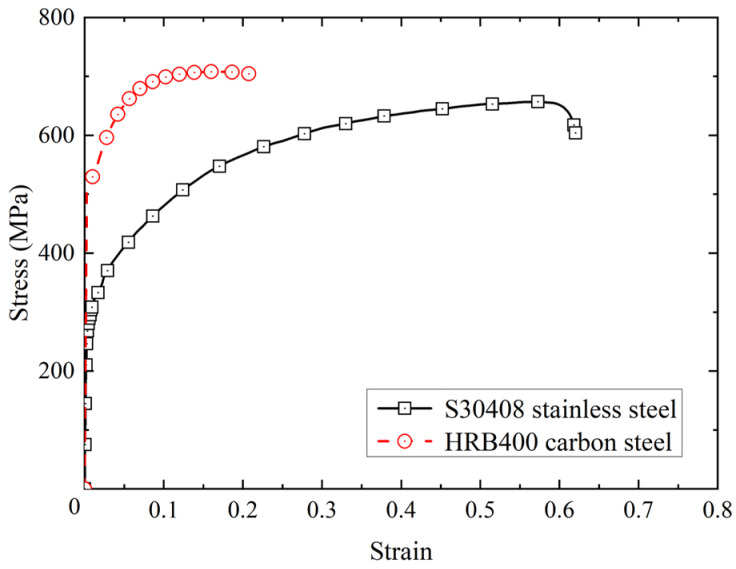
Mechanical properties of the stainless and carbon steels.

**Figure 21:**
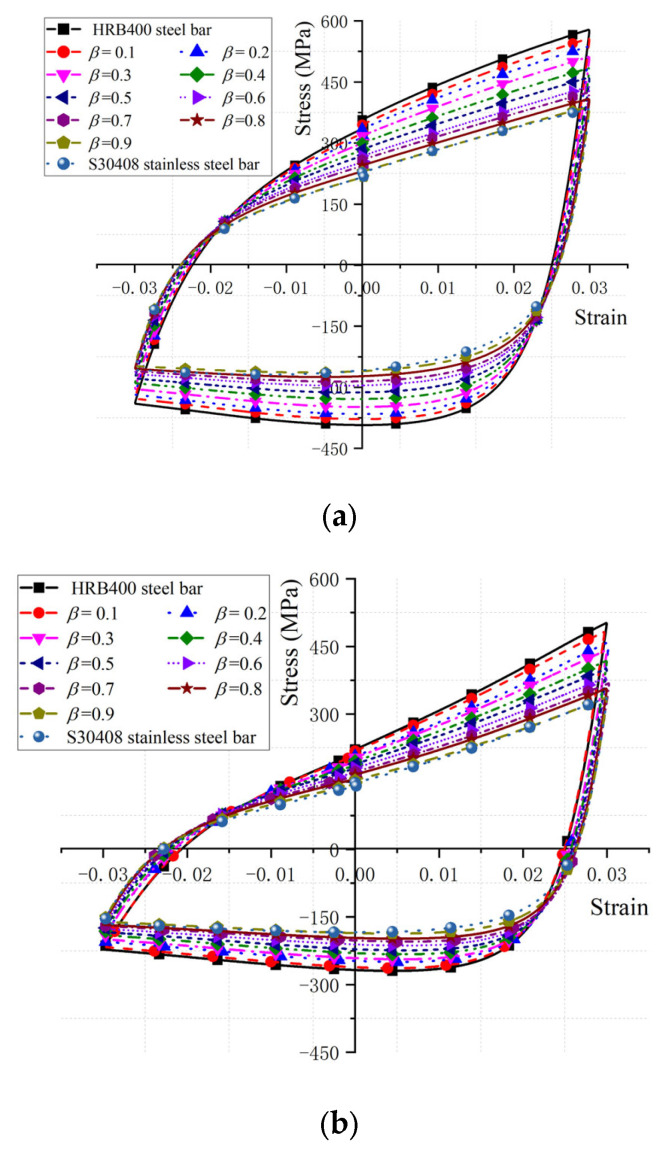

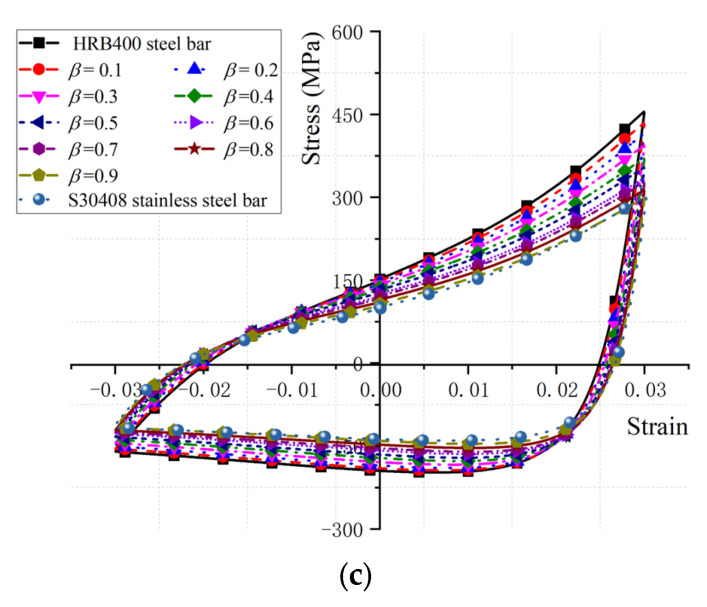
Hysteretic loop corresponding to 20% *N_f_*: (**a**) *L*/*D* = 9; (**b**) *L*/*D* = 12; (**c**) *L*/*D* = 15.

**Figure 22:**
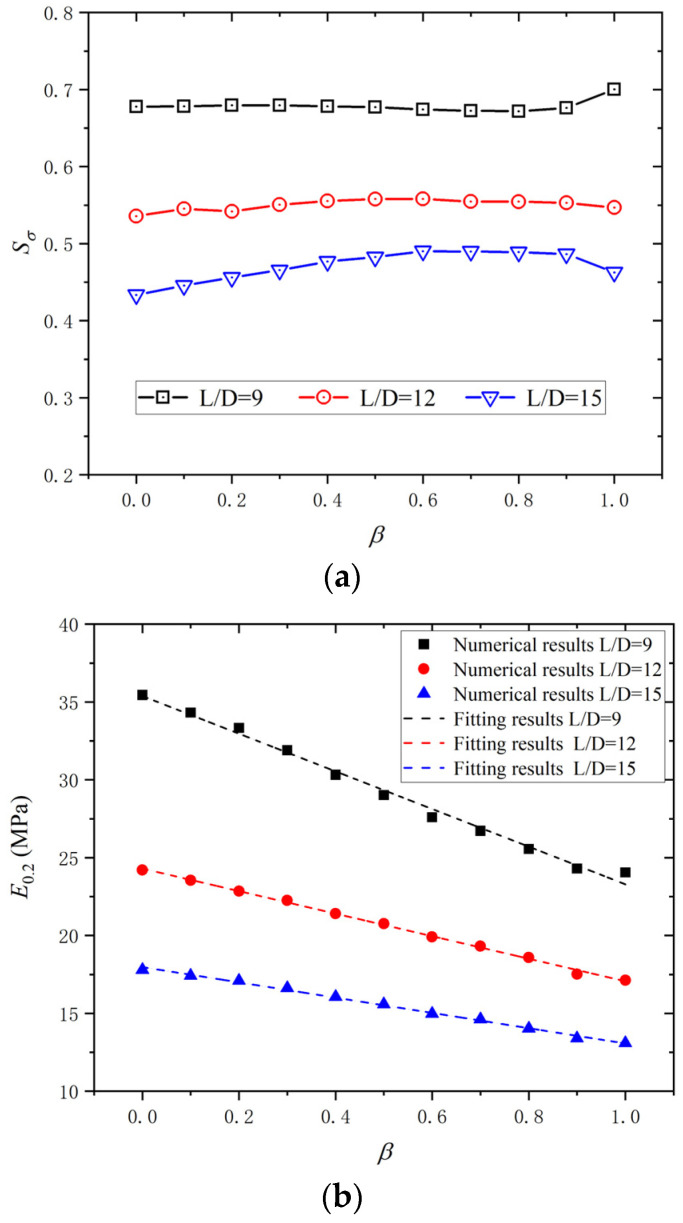
Influences of *β* on dissipate energy properties: (**a**) *S_σ_*; (**b**) *E*_0.2_.

The cladding ratio *β* of a BSB refers to the ratio of stainless-steel cladding area to the BSB cross-sectional area. Because of the significant differences in the mechanical properties between stainless steel and carbon steel, the *β* is an important factor influencing the monotonic mechanical property and LCF properties of the BSB. According to the numerical model proposed in Section 3.1, the effect of *β* on the LCF properties of BSBs was studied. Figure 20 shows a monotonic stress–strain relationship between carbon steel and stainless steel. The monotonic stress–strain data of BSBs with various *β* values were obtained from finite element results, which have been verified in reference [42]. Between 0% stainless steel (i.e., HRB400 steel bar) and 100% stainless steel (i.e., stainless steel bar), nine various *β* values were set for numerical analysis. According to the numerical analysis results, the hysteresis loop corresponding to a BSB with 20% *N_f_* is shown in Figure 21. With increases in *β*, the BSB peak stress decreased gradually. When the *L*/*D* and *ε*_a_ were the same, the higher the proportion of stainless steel, the lower the strength of the BSB. To clarify the relationship between the peak stress and the *β*, the *S_σ_* values of BSBs with different *β* values were statistically calculated, as shown in Figure 22a. It can be seen that the *S_σ_* values of BSBs with different *β* values tended to be stable at the same *L*/*D* and *ε*_a_. This evolution law illustrated that *β* has the same effect on the *σ*_c,max_ and *σ*_p,max_ of BSBs. With an increase in *β*, the *σ*_c,max_ and *σ*_p,max_ decrease synchronously. The hysteresis loop energy density (*E*_0.2_) corresponding to a BSB with 20% *N_f_* was calculated to quantify the dissipated energy properties of BSBs (Figure 22b). With an increase in *β*, the *E*_0.2_ reduced linearly. Equation (8) was proposed to quantify the relationship between *E*_0.2_ and *β*. The fitting results are shown in Table 4. It is recommended that the proportion of stainless steel in the BSB should be minimized once the corrosion resistance requirements are met.

(8) $$E_{0.2}=m+n\times \beta$$

## 4. Conclusions

Buckling significantly affected the LCF performance of the BSBs, which resulted in differences in the dissipated energy properties. In this study, an energy-based study on the LCF property of a BSB considering buckling was conducted using experimental and numerical methods. The main work and conclusions of this article are as follows:

(1) An experiment including five different *L*/*D* and three different *ε_a_* values was conducted to quantify the influences of buckling on the LCF properties of BSBs. The typical buckling mode of the BSBs was determined and three plastic hinges were observed. The impacts of the *L*/*D* and *ε_a_* on the *h_u_*/*L_u_* were revealed.
(2) The hysteretic loops of BSBs with various *L*/*D* and *ε_a_* values were compared. The hysteresis loops of the BSBs with *L*/*D* = 6 and *ε_a_* = 0.01 exhibited original symmetry. With increases in the *L*/*D*, the original symmetry of the hysteresis loop gradually disappeared owing to buckling. Test results stated that the *L*/*D* and *ε_a_* exhibited a coupling effect on the cycles to failure of the BSBs. The variation trends of the dissipated energy density and energy dissipation coefficient *R_d_* of the BSBs were discussed to clarify the effects of buckling.
(3) A numerical modelling method was suggested, which was carefully validated. The numerical results revealed that the influence of the number of elements on the simulation results was negligible. A predictive equation, which considered the effects of the *L*/*D*, was proposed to reveal relations between *ε_a_* and *N_f_*. With increases in the *L*/*D*, a pinching phenomenon of the hysteresis loop corresponding to 20% *N_f_* was gradually revealed. The *σ*_c,max_ and *σ*_p,max_ were reduced by increasing the *L*/*D*.
(4) For BSBs with various *ε_a_* values, the hysteretic loops corresponding to 20% *N_f_* shared a similar shape. Increases in *β* reduced the peak stress and dissipated energy properties of BSBs, so it is recommended that the proportion of stainless steel in BSBs should be minimized once the corrosion resistance requirements are met. Furthermore, *S_σ_* and *R_d_* were selected to determine the impacts of the *L*/*D* and *ε_a_* on the stress and dissipated energy properties.

In general, this study provides an experimentally verified numerical method to investigate the effects of buckling on the dissipated energy properties and LCF capacity of BSBs, which is meaningful for predicting the seismic resistant performance of reinforced concrete structures with BSBs. 

## Figures and Tables

**Table 1:** Chemical compositions of stainless steel and carbon steel.

Category (%)	C	Si	Mn	P	S	Ni	Cr	Cu	Al	Mo
S30408	0.201	0.421	1.441	0.008	0.004	0.047	0.087	0.045	0.014	0.009
HRB400	0.050	0.415	1.005	0.012	<0.001	8.230	18.410	0.071	0.002	0.024

**Table 2:** The specimen dimensions.

Number	*L*/*D*	Test Segment (mm)	Clamped Length (mm)	Full Length (mm)
B-6	6	108	80	268
B-9	9	162	80	322
B-12	12	216	80	376
B-15	15	270	80	430

**Table 3:** Peak stress of the BSB specimen.

Number	*L*/*D*	*ε_a_*	Peak Tensile Stress (MPa)	Peak Compressive Stress (MPa)
B-6-0.01	6	0.01	462.28	518.50
B-6-0.02	6	0.02	550.47	554.93
B-6-0.03	6	0.03	570.22	547.50
B-9-0.01	9	0.01	495.39	521.48
B-9-0.02	9	0.02	522.24	423.74
B-9-0.03	9	0.03	526.83	323.10
B-12-0.01	12	0.01	497.49	367.55
B-12-0.02	12	0.02	507.45	270.09
B-12-0.03	12	0.03	506.42	201.53
B-15-0.01	15	0.01	500.97	296.09
B-15-0.02	15	0.02	499.32	172.42
B-15-0.03	15	0.03	520.70	142.76

**Table 4:** Coefficient of fitting model.

*L*/*D*	*m*	*n*	Coefficient of Determination (R^2^)
9	35.38	−12.09	0.990
12	24.30	−7.24	0.998
15	17.97	−4.91	0.995

## Data Availability

The original contributions presented in the study are included in the article, further inquiries can be directed to the corresponding author.
